# Reducing the effects of control materials based on interchangeability of estimates of day‐to‐day imprecision between commercial control materials and serum samples

**DOI:** 10.1002/jcla.23710

**Published:** 2021-01-23

**Authors:** Qin Xie, Yi Tang, Meihua Zhou, Bing Dai, Xiaomin Zhao, Yating Cheng, Jun He, Chenli Zhang, Xiaoyan Deng, Lishi Li, Ran Tao

**Affiliations:** ^1^ Laboratory Diagnosis Department Changsha Kingmed Center for Clinical Laboratory Changsha China; ^2^ Laboratory Diagnosis Department Guangzhou Kingmed Center for Clinical Laboratory Guangzhou China; ^3^ KingMed School of Laboratory Medicine Guangzhou Medical University Guangzhou China; ^4^ Laboratory Diagnosis Department Taiyuan Kingmed Center for Clinical Laboratory Taiyuan China

**Keywords:** control material, imprecision, interchangeability, serum sample

## Abstract

**Background:**

Reduce the effects in the storage‐and‐thawing process of commercial control materials based on their interchangeability evaluation.

**Methods:**

Seven assays—anti‐streptolysin O, complement 3, carcinoembryonic antigen, urea, ferritin, total bilirubin, and glucose—were selected. Commercial control materials and serum samples with similar concentrations were chosen as samples. The experiment was carried out in three stages. In the first stage, the assays with statistical differences in imprecision were screened. In the second stage, two specimens were sealed with parafilm and frozen at −80°C and thawed in the water bath, and the imprecision differences were compared again. Finally, the effective means to reduce the effects were included in the standard operating procedure to repeat confirmation.

**Results:**

In the first stage, there was only a statistical difference (*p* < 0.05) in the imprecision of glucose and total bilirubin between two specimens, and the imprecision of control materials was higher than the serum samples. In the second stage, glucose imprecision was not statistically different (*p* > 0.05) and lower than in the first stage. In the third stage, the methods from the second stage were confirmed to be effective at reducing control material effects.

**Conclusion:**

Finding variation factors and confirming and standardizing the measures will help lessen commercial control material effects.

## Graphical abstract

Strictly controlling the operation process can be adopted to reduce the effects of control materials, so that the imprecision across commercial control materials and patient serum samples can be interchangeable.

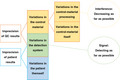

## 1 INTRODUCTION

When a clinical laboratory tests serum samples, it usually only conducts a single test and sends out a report. Therefore, it is important to control the imprecision of assays.^1^, ^2^ Estimations of day‐to‐day imprecision are usually made with control materials. In practice, however, the detection of a significant change between two consecutive results of an analyte in a patient requires knowledge of the day‐to‐day imprecision associated with patient results.^1^ The imprecision of patient results generally includes the biological variation of the patient themself and the analytical variation of the detection system. The purpose of quality control (QC) in the laboratory is to detect the analytical variation as far as possible and control it within a reasonable range, in order to ensure that the patient results truly reflect the patient's status.

However, the imprecision detected in the internal quality control often includes the analytical variation of the detection system and the variation of the control material. The control material variation is equivalent to the interference signal, and the larger the proportion, the more difficult it is to accurately detect the analytic variation. Only by reducing the control material variation as much as possible can the detection signal be amplified, so that the QC results can more truly reflect the analytic variation of the detection system and the control material can play a real role.

Many years ago, several authors asserted that there may be a lack of interchangeability between commercial control materials and serum samples regarding day‐to‐day imprecision.^3^, ^4^ These differences may come from the control material itself (ie, the matrix effect) or from variations in the control material processing (including storage and reconstitution) which does not exist in the operation process of serum samples. Once the control material is selected, the matrix effect cannot be amended. Therefore, the interchangeability of day‐to‐day imprecision for commercial control materials and serum samples as the standard is extremely important for evaluating how to reduce variations in control material processing. This standard can fundamentally evaluate whether the control material detection conforms to the specification; only when the imprecision between control materials and serum samples was interchangeable can all internal quality‐control behaviors be considered effective for serum samples. To our knowledge, however, no study has used this criterion to evaluate how to reduce the difference in imprecision between control materials and serum samples; and no study has added the improvements to the laboratory standard operating procedure to confirm whether the improvement could be repeated.

In addition, if the noninterchangeability were found among control materials from different manufacturers or, worse still, among different lots of the same control material, monitoring day‐to‐day imprecision during long periods also would be very difficult.^4^ Consequently, it is very important to strive to make the control materials have the imprecision interchangeable with the human samples.

In the present study, we compared the imprecision between commercial control materials and serum samples of seven assays of anti‐streptolysin O (ASO), complement 3 (C3), carcinoembryonic antigen (CEA), urea (UREA), ferritin (FER), total bilirubin (TBIL), and glucose (GLU), and we tried to reduce the effects of commercial control materials by referring to the experience of reference‐measurement research and discussing the feasibility of the method in the laboratory.

## 2 MATERIALS AND METHODS

### 2.1 Materials

Commercial control materials were purchased from Cliniqa Corp. (Liquid QC ImmuTROL Serum Protein Control, CA, USA) and Bio‐Rad Laboratories, Inc. (Lyphochek Assayed Chemistry Control, Lyphochek Tumor Marker Plus Control, and Lyphochek Immunoassay Plus Control, CA, USA). Serum samples are from routine patient samples with similar values obtained in the control materials, which were attained from the Changsha KingMed Center for Clinical Laboratory. Before analysis, specimens were stored at 2–8°C.

Anti‐streptolysin O and C3 were performed on the Cobas 6000 Analyzer Series (c501) (Roche Diagnostics), CEA was performed on the Cobas 6000 Analyzer Series (e601) (Roche Diagnostics), and FER was performed on the Architect System i2000sr (Abbott Laboratories); corollary reagents and calibrators were used in these three measurements. The remaining measurements were performed with the Model 7600 Series Automatic Analyzer (Hitachi High‐Technologies) with Maccura reagents and calibrators. The parafilm was purchased from Bemis Company, Inc.

### 2.2 Specimen processing and analysis

#### 2.2.1 Stage 1

Screening assays with a statistical difference in imprecision between commercial control materials and serum samples.

##### Sub‐package and storage

According to the manufacturer's specifications, the Liquid QC ImmuTROL Serum Protein Control for the ASO and C3 assays was not aliquoted and stored at 2–8°C until measurement; the other control materials were reconstituted previously; then, each reconstituted control material and each serum sample were divided into 20 aliquots and stored at −20°C away from light until analysis.

##### Thawing

Each vial of Liquid QC ImmuTROL Serum Protein Control was mixed upside‐down eight times before sampling to ensure homogeneity; then, the cap was immediately replaced and it was stored at 2–8°C. The samples were sealed and left at room temperature (25 ± 5°C) for 15 minutes. Each vial of samples that was frozen at −20°C was thawed at room temperature (25 ± 5°C) for 15 minutes. All samples were thoroughly mixed with pipettes and measured within 10 minutes.

##### Analysis

The control materials were analyzed first. After each assay was in control, one measurement of each analyte was carried out in each of the serum samples within 2 hours by the same analyst. The specimens were analyzed for 20 consecutive days. When 20 replicated results for each analyte were obtained, the corresponding variances and coefficients of variation (CVs) representing imprecision were estimated. The imprecision for each assay between the control materials and the serum samples was compared, and the assays with statistical differences were selected for the second phase of the experiment.

#### 2.2.2 Stage 2

Re‐comparing after improving the operational procedures of assays with differences in Stage 1.

Two serum samples were re‐collected and pooled into a plain tube. After thoroughly mixing, each pool was aliquoted into 0.5‐ml Eppendorf tubes. Then, each pool was composed of 40 aliquots, and the samples were randomly divided into two experimental groups with 20 aliquots in each group. The samples of Group 1 were stored, thawed, and measured as the first phase of the experiment. The samples of Group 2 were sealed with parafilm and stored at −80°C away from light until analysis. Thirty minutes before analysis, one aliquot of the samples of Group 2 was removed from −80°C, thawed in the water bath (25 ± 2°C) away from light for 10 minutes, mixed gently upside‐down five times, left at room temperature (25 ± 5°C) away from light for 15 minutes. It was mixed gently upside‐down again for five times and then measured within 10 minutes.^5^, ^6^, ^7^, ^8^, ^9^ Commercial control materials were processed as serum samples. After each assay was in control each day, over the course of 20 working days, one measurement of each analyte was carried out in each of the control materials and serum samples simultaneously by the same analyst.

#### 2.2.3 Stage 3

Verify that the operational improvements in Stage 2 are reproducible.

Only control materials were analyzed, and the analyses were expanded to 21 analytes, and then, the difference in imprecision between the two specimen‐processing methods for 20 days was compared.

### 2.3 Statistical analysis

The mean, standard deviation (SD), and CV for each assay were calculated to compare the imprecision. When the ratio of mean‐to‐SD was less than 3, the SDs of the replicate analyses were compared by the *F*‐test, where *F* = (SD_1_)^2^/(SD_2_)^2^ and SD_1_ > SD_2_. Otherwise, the CVs were compared by a modification of the *F*‐test, which has been designated the *H*‐test, where *H* = (CV_1_)^2^/(CV_2_)^2^ and CV_1_ > CV_2_. The *F* Bilateral Boundary Table, F_0.05(19,19)_ = 2.51–2.62, was queried; if *F* or *H* was greater than 2.62, it was regarded as significant (*p* < 0.05).^3^ Additionally, imprecision was compared with desirable analytical‐quality specifications for imprecision upon biological variation.^10^ The biological variation data preferentially used the latest data from the European Federation of Clinical Chemistry and Laboratory Medicine (EFLM).^11^

## 3 RESULTS

Each pair of variances was compared by the *H*‐test, because the ratios of the means‐to‐SDs were greater than 3.

**FIGURE 1:**
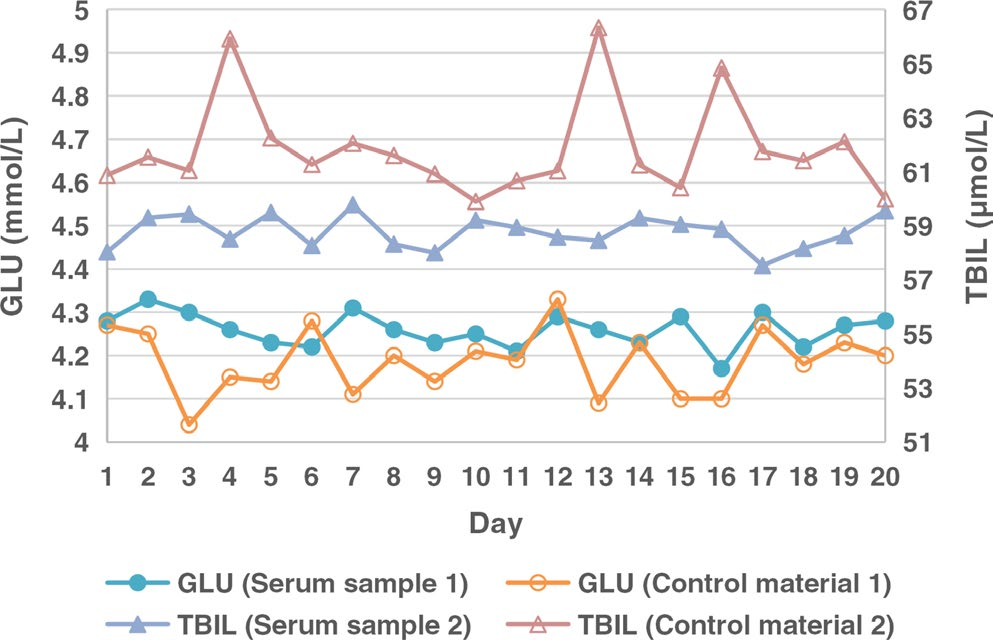
Comparison of imprecision between commercial control materials and serum samples of GLU and TBIL analyses. GLU, glucose; TBIL, total bilirubin

Results of the comparisons in the first stage are shown in Table 1. There is no specification for imprecision for ASO, because it does not have biological variation data. Except C3, other assays met the desirable specifications for imprecision. There was only a statistical difference in the imprecision of GLU (Level 1) and TBIL (Level 2) between commercial control materials and serum samples. Figure 1 shows the arrangement of serum sample data and QC data of GLU (Level 1) and TBIL (Level 2) within 20 days. Each group of data fluctuated above and below the respective mean, and there was no obvious trend change.

**TABLE 1:** Comparison of the imprecision between two specimen types of seven assays

Analyte	Specifications for imprecision (%)	Level	Serum samples			Control materials			*H*‐test
			Mean	SD	CV (%)	Mean	SD	CV (%)	
ASO, IU/ml	n/a	1	129.60	2.503	1.93	127.30	2.627	2.06	NS
		2	274.00	4.807	1.75	346.60	7.121	2.05	NS
C3, g/L	2.3	1	1.31	0.0429	3.28^a^	0.74	0.0215	2.91^a^	NS
		2	2.83	0.0938	3.31^a^	2.31	0.0730	3.16^a^	NS
CEA, ng/ml	9.0	1	3.91	0.118	3.02	3.95	0.0990	2.51	NS
		2	50.03	0.750	1.50	67.62	1.157	1.71	NS
GLU, mmol/L	2.5	1	4.26	0.0397	0.93	4.19	0.0758	1.81	*p* < 0.05
		2	14.53	0.151	1.04	14.38	0.184	1.28	NS
TBIL, μmol/L	11.9	1	12.82	0.170	1.33	14.89	0.277	1.86	NS
		2	58.77	0.615	1.05	61.86	1.789	2.89	*p* < 0.05
UREA, mmol/L	7.0	1	5.29	0.125	2.37	5.34	0.138	2.59	NS
		2	16.28	0.391	2.40	15.92	0.446	2.80	NS
FER, ng/ml	6.4	1	71.69	2.130	2.97	70.89	1.818	2.56	NS
		2	489.44	14.141	2.89	463.34	11.187	2.41	NS

Abbreviation: NS, Not significant (*p* > 0.05).

^a^ Assays did not meet the desirable specifications for imprecision.

For the second stage, the comparison results of the imprecision of the GLU analyses between two specimen types with two different processing methods are shown in Table 2. The CVs of the analyses of two specimens with two different processing methods met the desirable specifications for imprecision. Under the condition, “stored at −20°C and thawed at room temperature,” there was a statistical difference in the imprecision between commercial control materials and serum samples; there was no statistical difference in the imprecision between two specimen types under the condition, “sealed with parafilm, frozen at −80°C, and thawed in water bath.” In addition, there was no statistical difference in the imprecision of serum samples under different processing methods, while the control material variations under “sealed with parafilm, frozen at −80°C, and thawed in water bath” was less than under “stored at −20°C and thawed at room temperature,” and the difference was statistically significant.

**TABLE 2:** Comparison of the imprecision of GLU analyses of two specimen types with two different processing methods

Groups	Serum samples			Control materials			*H*‐test
	Mean (mmol/L)	SD (mmol/L)	CV (%)	Mean (mmol/L)	SD (mmol/L)	CV (%)	
G1: stored at −20°C and thawed at room temperature	3.79	0.0415	1.09	3.91	0.0733	1.88	*p* < 0.05
G2: sealed with parafilm, frozen at −80°C, and thawed in water bath	3.84	0.0287	0.75	4.02	0.0453	1.13	NS
*H*‐test	/	/	NS	/	/	*p* < 0.05	/

Abbreviation: NS, Not significant (*p* > 0.05).

**FIGURE 2:**
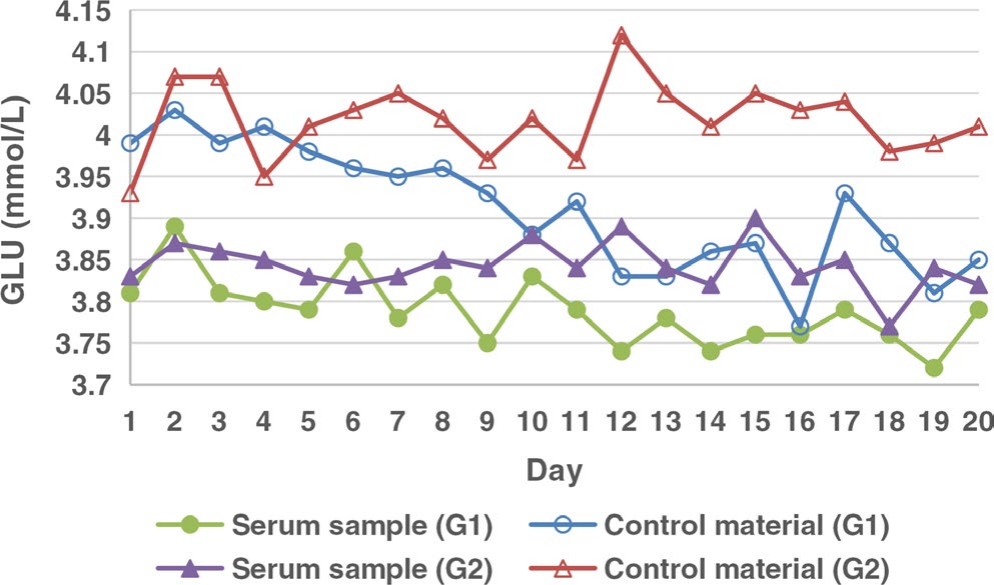
Comparison of the imprecision of the GLU analyses of two specimen types with two different processing methods. GLU, glucose

Meanwhile, the trend changes of the four groups of data in the second stage are shown in Figure 2. According to the detection values, both the serum samples and the commercial control materials under the condition, “stored at −20°C and thawed at room temperature,” showed a significant decreasing trend with the extension of the days, especially for the commercial control materials, the decline was close to 4%, exceeding the desirable analytical‐quality specifications for imprecision upon biological variation.

In the third stage, the comparison results of the imprecision of two different processing methods for 21 assays are shown in Table 3. Among the 42 concentration levels of the 21 assays, 35 concentration levels showed that the control material imprecision under “sealed with parafilm, frozen at −80°C, and thawed in water bath” was less than under “stored at −20°C and thawed at room temperature”; of these, the differences were statistically significant in 10 concentration levels of eight assays, and the differences were all statistically significant in two concentration levels for creatinine (CREA) and lactate dehydrogenate 1 (LDH 1). In addition, among the assays with statistical difference in the above comparison, only under the condition, “stored at −20°C and thawed at room temperature,” the analyses of LDH 1 (two concentration levels) and HDL cholesterol (Level 2) did not meet the desirable analytical‐quality specifications for imprecision upon biological variation. There is no specification for imprecision for α‐hydroxybutyrate dehydrogenase, because it does not have biological variation data.

**TABLE 3:** Comparison of the imprecision of two different processing methods for the Lyphochek Assayed Chemistry Control

Analyte	Specifications for imprecision (%)	Level	−20°C			−80°C			*H*‐test
			Mean	SD	CV (%)	Mean	SD	CV (%)	
Glucose, mmol/L	2.5	1	3.92	0.039	1.00	4.05	0.028	0.69	NS
		2	14.28	0.103	0.72	14.54	0.082	0.56	NS
Creatine kinase, U/L	7.5	1	118.65	1.954	1.65	116.90	1.483	1.27	NS
		2	430.00	7.269	1.69	426.75	3.611	0.85	*p* < 0.05
Phosphate, mmol/L	4.1	1	1.15	0.013	1.12	1.16	0.015	1.28	NS
		2	2.36	0.027	1.13	2.39	0.020	0.83	NS
α‐hydroxybutyrate dehydrogenase, U/L	n/a	1	155.55	3.720	2.39	155.05	2.856	1.84	NS
		2	380.45	7.640	2.01	381.65	5.393	1.41	NS
Albumin, g/L	1.3	1	40.90	0.448	1.10	40.74	0.484	1.19	NS
		2	27.36	0.395	1.44^a^	27.34	0.384	1.41^a^	NS
Alkaline phosphatase, U/L	2.7	1	80.90	2.426	3.00^a^	81.40	2.210	2.72^a^	NS
		2	351.75	10.29	2.93^a^	344.20	6.254	1.82	NS
Alanine aminotransferase, U/L	9.7	1	29.90	0.788	2.64	30.90	0.968	3.13	NS
		2	97.90	1.411	1.44	97.90	1.210	1.24	NS
Aspartate aminotransferase, U/L	6.2	1	42.00	1.622	3.86	44.85	1.137	2.53	NS
		2	213.40	2.088	0.98	216.20	1.436	0.66	NS
Creatinine, μmol/L	2.3	1	149.10	2.864	1.92	148.30	1.418	0.96	*p* < 0.05
		2	474.30	7.678	1.62	477.20	2.462	0.52	*p* < 0.05
Bilirubin, conjugated, μmol/L	18.4	1	6.19	0.213	3.45	6.29	0.234	3.72	NS
		2	18.10	0.354	1.96	18.76	0.323	1.72	NS
γ‐Glutamyltransferase, U/L	6.7	1	53.65	0.745	1.39	53.15	0.489	0.92	NS
		2	149.45	1.669	1.12	146.90	1.619	1.10	NS
Lactate dehydrogenate 1, U/L	1.2	1	79.24	1.807	2.28^a^	79.82	0.924	1.16	*p* < 0.05
		2	249.01	3.931	1.58^a^	250.31	2.381	0.95	*p* < 0.05
Lactate dehydrogenate, U/L	4.3	1	160.25	3.810	2.38	160.05	2.911	1.82	NS
		2	363.50	8.023	2.21	362.95	5.052	1.39	NS
HDL cholesterol, mmol/L	2.9	1	1.56	0.028	1.82	1.53	0.017	1.13	NS
		2	0.67	0.021	3.21^a^	0.64	0.006	0.94	*p* < 0.05
LDL cholesterol, mmol/L	4.2	1	3.20	0.080	2.50	3.16	0.075	2.37	NS
		2	1.47	0.041	2.76	1.45	0.032	2.24	NS
Bilirubin, total, μmol/L	11.9	1	15.31	0.193	1.26	15.61	0.157	1.00	NS
		2	66.74	0.674	1.01	67.30	0.415	0.62	*p* < 0.05
Cholesterol, mmol/L	2.7	1	6.48	0.048	0.74	6.47	0.039	0.61	NS
		2	2.70	0.047	1.74	2.68	0.026	0.98	*p* < 0.05
Triglyceride, mmol/L	10.0	1	2.02	0.016	0.80	2.02	0.014	0.67	NS
		2	1.02	0.012	1.15	1.02	0.007	0.70	*p* < 0.05
Protein, total, g/L	1.3	1	62.51	0.785	1.26	64.10	0.890	1.39^a^	NS
		2	41.39	0.768	1.86^a^	42.40	0.775	1.83^a^	NS
Urate, μmol/L	4.3	1	278.85	1.226	0.44	278.25	1.118	0.40	NS
		2	575.60	4.684	0.81	574.90	1.832	0.32	*p* < 0.05
Urea, mmol/L	7.0	1	5.37	0.123	2.29	5.47	0.140	2.55	NS
		2	16.15	0.365	2.26	16.34	0.371	2.27	NS

Abbreviation: NS, Not significant (*p* > 0.05).

^a^ Assays did not meet the desirable specifications for imprecision.

## 4 DISCUSSION

The previous studies have mainly focused on the stability of serum samples and control materials or the interchangeability of day‐to‐day imprecision for them.^3^, ^4^, ^5^, ^6^, ^7^, ^8^, ^9^ In this study, we screened out assays with differences in imprecision between the commercial control materials and the serum samples, and made improvements based on the performance of these assays. After confirming the effect, the improvements were added to the laboratory standard operating procedure to confirm whether the improvements could be repeated.

**FIGURE 3:**
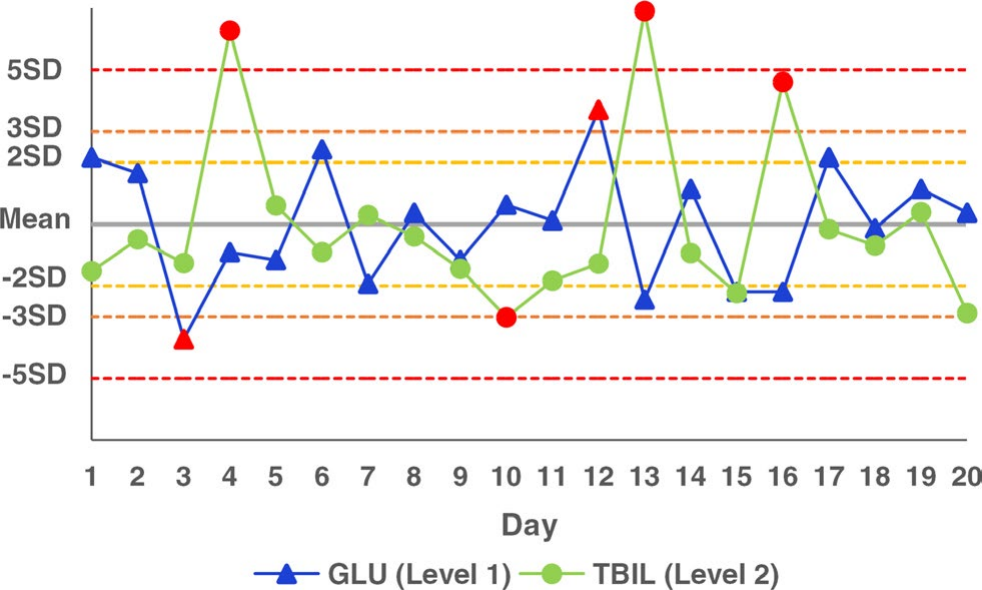
*Z*‐value control chart of GLU and TBIL based on the mean of the control material and the SD of the serum sample. GLU, glucose; TBIL, total bilirubin

In the first stage of this study, the imprecision between two specimen types was compared by using the daily operating procedures in our laboratory, of which the differences of GLU (Level 1) and TBIL (Level 2) were statistically significant, and the SDs obtained by analyses of commercial control materials were 1.9 times and 2.9 times that of serum samples, respectively. Although their imprecision meets desirable analytical‐quality specifications for imprecision upon biological variation, if the mean of the control material and the SD of the serum sample are used for plotting control chart (Figure 3), in the case of no systematic error, there are two QC points in GLU and four QC points in TBIL which go out of a three SD‐interval and their probabilities are as high as 10% and 20%, respectively. In practice, however, the SD of the control material is taken as the control interval, and there are no QC points which go out of a three SD‐interval. Therefore, the control material variation increases the control interval and the out‐of‐control probability increases, which increases the laboratory's verification cost for false out of control. If the influence of control material variation can be reduced, the QC data can provide more useful information of the detection system and reduce the QC cost. In addition, other assays and concentrations in the first stage, including GLU (Level 2), TBIL (Level 1), FER, UREA, ASO, C3, CEA, showed no statistical difference in the imprecision between two specimen types.

In 2002, Fuentes‐Arderiu et al^4^ compared the imprecision between commercial control materials (Bio‐Rad) and serum samples, but the only results that were similar to this study were for UREA. The difference was due to the imprecision of the analyses of commercial control materials at two concentration levels for GLU, TBIL, and FER, which were statistically different from the serum samples, and the imprecision of the GLU and TBIL analyses in commercial control materials was less than what was obtained with serum samples. This shows that there are many factors that affect quality‐control efficiency, including the control materials themselves, operators, operating methods. The best way to improve quality‐control efficiency may be to strictly control the operating process to reduce the variation in control material processing.

In addition to the matrix effect, the likely causes of the statistical difference in the imprecision between the commercial control materials and the serum samples include^3^, ^4^, ^6^ (1) variations in the preparation and reconstitution of the control material (ie, the variations between bottles); (2) differences in the stability of the two samples, which affected factors that included sample moisture evaporation, storage temperature, the freeze‐thaw process; (3) insufficient sample mixing.

In the second stage, we selected several of the above factors for standardized operation: low temperature, anti‐evaporation, and gradient thawing (ie, the thawing method of Group 2 in Stage 2). Parafilm seal can prevent sample moisture evaporation, while low temperature and gradient thawing can reduce analyte damage during the preservation and thawing process; that is, the influence of “cold denaturation.” The results showed that there was no statistical difference in the imprecision between the commercial control materials and the serum samples when the operation process was strictly controlled, which indicates that this method could effectively reduce the imprecision difference between two specimen types. At the same time, the results also showed that the uncontrolled specimens are more imprecise than strictly controlled specimens, and the measured data showed a decreasing trend, which was mainly affected by the so‐called “cold denaturation.” This result was consistent with the literature reports.^5^, ^6^, ^7^, ^8^, ^12^

In order to confirm whether the operation can be extended to other assays, we incorporated the operation in the conclusion into the laboratory standard operating procedure and applied it to 21 routine assays. The results showed that there was a statistically significant decrease in the imprecision of eight assays, and the decrease in the imprecision of enzymes and micromole‐level analytes was more obvious, and the imprecision of LDH 1 and HDL cholesterol (Level 2), which originally did not meet the specification, met the standard, which indicates the extendibility of the operation.

The limitation of this paper is that in the second stage, due to the difficulty of collecting serum samples, the comparison experiment of only the glucose analyses was carried out. In addition, in the control material operation, other standardized operations used in previous studies, such as adding samples with dilution dispenser and using water with different conductivity (whether <1 μs/cm), were not included because of the limitations of the experimental conditions.

Although the main result found in this study, “some measurands, especially glucose, are unstable if stored at −20°C instead of −80°C,” it is well known, but because of the cost, customary and convenience, the laboratories generally keep the control materials at −20°C (recommended by the manufacturer) instead of −80°C. This paper explains its necessity from the perspective of improving the interchangeability of day‐to‐day imprecision for control materials and serum samples.

In summary, by comparing the analytical imprecision between control materials and serum samples, we can select a control material that has the imprecision interchangeable with the patient sample as much as possible. When selecting, attention should be paid to those assays with great coefficients of variation and poor interchangeability. If there are still assays with poor interchangeability with patient samples in the selected control materials, the method of strictly controlling the operation process in this study can be adopted to reduce the effects of control materials, so that the imprecision across control materials and patient serum samples can be interchangeable.

## CONFLICT OF INTEREST

The authors declare that they have no known competing financial interests or personal relationships that could have appeared to influence the work reported in this paper.

## Data Availability

The data that support the findings of this study are available from the corresponding author upon reasonable request.
